# Use of Ozone Therapy and Thymoquinone in the Prevention of Formaldehyde Toxicity by Inhalation: An Experimental Study

**DOI:** 10.7759/cureus.54914

**Published:** 2024-02-26

**Authors:** Feyza Aksu, Ahmet Kavaklı, Tuncay Kuloglu, Seval Yilmaz, Emre Kaya, Ramazan Fazil Akkoc, Mustafa Yilmaz, Elif Emre, Murat Ogetürk

**Affiliations:** 1 Department of Anatomy, Faculty of Medicine, Firat University, Elazig, TUR; 2 Department of Histology and Embryology, Faculty of Medicine, Firat University, Elazig, TUR; 3 Department of Biochemistry, Faculty of Veterinary Medicine, Firat University, Elazig, TUR; 4 Department of Emergency Medicine, Faculty of Medicine, Firat University, Elazig, TUR

**Keywords:** tunel, ozone therapy, thymoquinone, liver, formaldehyde exposure

## Abstract

Introduction: The study determined the damage caused by formaldehyde (FA) exposure in blood and liver samples using biochemical markers. Histopathological analysis was performed using the terminal deoxynucleotidyl transferase dUTP nick end labeling (TUNEL) method and measurement of CD68 cell density. To what extent the antioxidant molecules thymoquinone (TQ) and ozone (O_3_) reversed the damage caused by FA exposure was investigated, both when used alone and combined.

Methods: Fifty-six Sprague-Dawley male rats of eight to ten weeks of age were used in the experiment. The rats were divided into eight groups, with seven rats in each group: the untreated control group, the group treated with TQ (10 mg/kg/day), the group treated with O_3 _(150 μg/kg/day), the group treated with TQ+O_3_, the group exposed to FA (10 ppm 8 h/day), the group receiving FA+TQ, the group receiving FA+O_3_, and the group receiving FA+TQ+O_3_. Serum aspartate transaminase (AST), alanine transaminase (ALT), total antioxidant (TAS, U/mL), and total oxidant (TOS, nmol/mL) levels were analyzed. TAS and TOS levels, CD68 cell density, and apoptotic cells were determined in liver tissues.

Results: FA exposure caused an increase in serum AST and ALT levels of (p<0.05) experimental animals, a decrease in TAS levels in serum (p=0.03) and liver (p>0.05) and an increase in TOS levels (p>0.05), TUNEL positivity (p<0.001), and CD68 cell density (p=0.004). Administration of TQ and O_3_ as antioxidants significantly reversed biochemical and histopathological alterations in the serum and liver.

Conclusion: TQ and ozone therapy suppressed oxidative stress caused by FA exposure and reversed the emerging histopathological deteriorations. Ozone therapy did not suppress the effects of TQ. Therefore, ozone therapy can be given as a supportive therapy along with the main therapeutic agents. We think TQ and ozone therapy may be useful to protect individuals exposed to FA.

## Introduction

Formaldehyde (FA) is a chemical substance found naturally in living organisms. It has a strong, pungent odor, is colorless, dissolves well in water, and is the simplest member of the aldehyde family [1]. FA is absorbed into the body through the respiratory and digestive systems and has been shown to cause damage to many systems in the body. The occupational groups such as anatomists, taxidermists, histologists, pathologists, and those engaged in FA production are the groups with increased FA exposure, and certain medical effects have been reported to be observed in these groups. Studies have shown that FA can damage various tissues, including the eyes, nose, upper respiratory tract, lungs, and liver, while also reducing the antioxidant capacity of these tissues. Various antioxidant molecules have been reported to help prevent or mitigate this damage [2-5].

Thymoquinone (TQ) is the most prevalent and pharmacologically effective component of *Nigella sativa* (*N. sativa*), known for its antioxidant effect. *N. sativa*, commonly known as black cumin, is an herbaceous plant from the Ranunculaceae family with slender leaves, and it can grow up to 40 cm tall [6,7]. It also inhibits free radical formation, suppresses microsomal lipid peroxidation, stimulates polymorphonuclear leukocytes, and protects against the harmful effects of oxygen [8].

Ozone (O_3_), a gaseous molecule with an unstable structure due to its three-oxygen content, is known to have antioxidant effects. Ozone is converted into oxygen in the body. The atom freed during the formation of oxygen from ozone exhibits its effect by binding to other existing molecules. Ozone treatment has been used in many diseases, primarily infectious, immune system, vascular, orthopedic, and degenerative diseases, since the discovery of the gas form in 1839. The therapeutic ozone administration dose range is between 5 and 60 μg/ml and divided into three concentrations: low (10-20 μg/ml), medium (20-30 μg/ml), and high concentration (30-60 μg/ml). It shows immunomodulatory effects at low concentrations, both immunomodulatory and antioxidant effects at medium concentrations, and anti-inflammatory effects at high concentrations. Medical ozone treatment is generally used as a complementary and adjunctive therapeutic agent in treatment protocols [9,10].

The objective of this study is to determine the effects of TQ, ozone therapy, and the combination of both in the prevention of inhalation FA toxicity by measuring total antioxidant level (TAS), total oxidant level (TOS), malondialdehyde (MDA), glutathione reductase (GSH), catalase (CAT), glutathione peroxidase (GPx), and superoxide dismutase (SOD) levels.

## Materials and methods

The study was commenced after obtaining the approval of the Firat University Animal Experiments Local Ethics Committee (2019-2019/5). Animal care and experimental procedures were performed per the National Institute of Health guidelines for the care and use of laboratory animals. The research involved the use of 56 Sprague-Dawley rats that were male, eight to ten weeks old, and weighed between 270 and 300 g. All rats were housed at 22±1°C in clean, quiet, and well-organized rooms on light and dark cycles lasting 12 hours each. All rats were given water and rat chow ad libitum. Animals were monitored daily [4]. The sample size was calculated using the sample size calculating software G*Power version 3.1.9.2 (Universität Kiel, Germany). With 80% power, a 0.05 level of statistical significance, and an effect size of 0.6, the sample size for each group was calculated to be seven. Rats were randomly divided into eight groups of seven. An online random number generator was used as an example of suitable randomization methods (https://www.graphpad.com/quickcalcs/randomize1/). The experiment lasted four weeks. Group 1 rats constituted the control group exposed to normal atmospheric oxygen. Group 2 rats were administered ozone therapy at 150 μg/kg/day intraperitoneally (IP) five days a week. Group 3 rats were administered TQ at 10 mg/kg/day IP five days a week [11]. Group 4 rats were administered 150 μg/kg/day IP ozone therapy and 10 mg/kg/day IP TQ five days a week, with an eight-hour interval between the two treatments. Group 5 rats were administered 10 ppm eight hours/day of FA via inhalation. Group 6 rats were administered 10 ppm eight hours/day of FA via inhalation and 150 μg/kg/day IP ozone therapy five days a week. Group 7 rats were administered 10 ppm eight hours/day of FA via inhalation and 10 mg/kg/day IP TQ five days a week. Group 8 rats were administered 10 ppm eight hours/day of FA via inhalation, 150 μg/kg/day IP ozone therapy five days/week, and 10 mg/kg/day IP TQ five days/week (Table 1). TQ in powder form was dissolved in 20% dimethyl sulfoxide and administered to each rat as an IP injection at a dose of 10 mg/kg [11].

**Table 1:** Groups, chemicals applied, and dosages. TQ: thymoquinone; FA: formaldehyde; O_3_:_ _ozone.

	FA	O_3_	TQ
Group 1 (control)	-	-	-
Group 2 (O_3_)	-	150 μg/kg/day	
Group 3 (TQ)	-	-	10 mg/kg/day
Group 4 (TQ+O_3_)	-	150 μg/kg/day	10 mg/kg/day
Group 5 (FA)	10 ppm 8 h/day	-	-
Group 6 (FA+O_3_)	10 ppm 8 h/day	150 μg/kg/day	-
Group 7 (FA+TQ)	10 ppm 8 h/day	-	10 mg/kg/day
Group 8 (FA+TQ+O_3_)	10 ppm 8 h/day	150 μg/kg/day	10 mg/kg/day

Experimental procedure

The environment where the experimental animals were housed and the FA inhalation procedure were performed according to the method previously reported by Aydin et al. [4]. Glass cages of 20×50×100 cm were used to house the rats. Two cotton balls were suspended from the top of the cage, and 1 ml of 10% FA was injected into the center of the cotton. The FA release within the cage was monitored for eight hours using a calibrated FA monitor (ZDL-300; Environmental Sensors Co., Boca Raton, FL). During the experiment, FA saturation was kept constant at 10.20±0.16 ppm. Twenty-four days after the experiment started, all rats were injected IP with 75 mg/kg ketamine under anesthesia and then sacrificed by decapitation.

Blood and liver samples were collected from the rats. The liver tissues were immersed in 10% FA for histological analysis. Liver tissue homogenates were centrifuged at 1,792×g for 30 min at 4°C, and the supernatant was distributed into Eppendorf tubes with 500 KIU aprotinin. The blood sample was divided into plain and ethylenediaminetetraacetic acid (EDTA) biochemistry tubes for biochemical analysis. Both tubes were centrifuged at 1,792×g, transferred to Eppendorf tubes, and stored at −20°C until analysis.

Ozone generation

Ozone (O_2_/O_3_ mixture) was produced from medical-grade oxygen with Ozonator equipment in a Humazona device (Humares, Germany), and ozone concentration was measured using a UV spectrophotometer at 254 nm. The ozone gas obtained was an oxygen-ozone mixture containing 95% oxygen and 5% ozone.

Biochemical analyses

The experiment was concluded by sacrificing the rats and extracting liver samples. Before analysis, liver tissues were washed with physiological saline, diluted 1:10 with distilled water, and homogenized in a Potter-Elvehjem homogenizer (CAT R50D, Germany). The homogenate was centrifuged at 4°C at 3,000 g for 15 min to quantify MDA, GSH, CAT, GST, and SOD and at 10,000 g for 55 min to test the amount of GSH-Px. MDA, GSH, CAT, GST, SOD, and GSH-Px activities were determined by spectrophotometers (GENESYS™ 10S UV-Visible Spectrophotometers, Thermo Scientific, Waltham, Massachusetts, USA).

Plasma was used to determine aspartate transaminase (AST) and alanine transaminase (ALT) activities with the use of an Advia 1800 Chemistry Analyzer (Siemens Healthineers, Germany).

Blood and tissue TAS (RL0017) and TOS (RL0024) levels were analyzed using the Human TOS and TAS ELISA Kit (Sunred Biological Technology Co. Ltd., Shanghai, China). Blood and tissue TAS values were measured as U/ml and TOS as nmol/ml.

Immunohistochemistry

Samples collected for CD68 immunoreactivity in liver tissues were placed in 10% neutral FA and processed through routine histologic follow-up series, and paraffin blocks were prepared. Sections 4-6 mm thick were cut from the prepared paraffin blocks and placed on polylysine slides. Sections were deparaffinized, rehydrated in alcohol, and then stained immunohistochemically using the avidin-biotin-peroxidase (ABC) method [12,13] and CD68 primary antibody (Rabbit CD68 Polyclonal Antibody, E-AB-40066, Elabscience, USA). The antibody was diluted 1:200 with phosphate-buffered saline (PBS). Sections were analyzed and photographed using a Leica DM500 microscope (Leica, Nussloch, Germany). A histoscore was generated based on the prevalence (0.1: <25%, 0.4: 26-50%, 0.6: 51-75%, 0.9: 76-100%) and severity (0: none, +0.5: very small, +1: small, +2: moderate, +3: strong) of immunoreactivity. Histoscore=prevalence × severity [14].

Terminal deoxynucleotidyl transferase dUTP nick end labeling (TUNEL) method

We followed the manufacturer's instructions for the ApopTag Plus Peroxidase In Situ Apoptosis Detection Kit (Chemicon, floor no. S7101, USA) and used TUNEL staining to identify apoptotic cells [15]. Deparaffinized tissues were washed with PBS, counterstained with Harris hematoxylin after a series of incubations, and then examined, evaluated, and photographed under a Leica DM500 microscope (Leica DFC295). Cells with brown nuclear staining were counted as apoptotic, while nuclei stained blue with Harris hematoxylin were considered normal in the TUNEL staining evaluation. In randomly selected areas of the sections at 10× magnification, at least 500 normal and apoptotic cells were counted. The apoptotic index was calculated by the ratio of apoptotic cells to total (normal+apoptotic) cells and analyzed statistically [16].

Statistical analysis

The IBM SPSS Statistics for Windows, Version 20 (Released 2011; IBM Corp., Armonk, New York, USA) was used for statistical analysis. Differences between group averages were calculated using the one-way analysis of variance. Duncan's test was used as a post-hoc test, and all values were expressed as mean±standard deviation of the mean (mean±SEM). p≤0.05 was considered statistically significant.

## Results

Clinical changes that developed in rats at the end of the study period

Yellowing was detected in the fur of rats exposed to FA. Rats exposed to FA inhalation showed slowing of movements, frequent nose clearing, excessive eye blinking, and increased licking. In addition, while the average weight of rats administered FA inhalation at the beginning of the study was 282 g, after four weeks, the average weight was 263 g, and a decrease in body weight was detected.

Laboratory findings

There was no significant difference between the blood AST and ALT levels of rats in the control group, O_3_ group, TQ group, and TQ+O_3_ group. Compared with the control group, we observed a significant increase in AST and ALT values in the FA group (p<0.05). AST and ALT levels decreased significantly in the groups receiving O_3_ or TQ or TQ+O_3_ with FA inhalation compared to the FA group, while there was no difference with the control group (Tables 2, 3).

**Table 2:** MDA, GSH, CAT, GPx, SOD, TAS, and TOS levels in the liver serum of rats exposed to the control group, O3 group, TQ group, TQ+O3 group, FA group, FA+O3 group, FA+TQ group, and FA+TQ+O3 group. ^a^Significantly different compared to the control group. MDA: malondialdehyde; GSH: glutathione reductase; CAT: catalase; GPx: glutathione peroxidase; SOD: superoxide dismutase; TAS: total antioxidant level; TOS: total oxidant level; TQ: thymoquinone; O_3_: ozone; FA: formaldehyde.

	Control	O_3_	TQ	TQ+O_3_	FA	FA+O_3_	FA+TQ	FA+TQ+O_3_
MDA (nmol/ml)	8.27±0.29	8.44±0.50	8.08±0.32	8.28±0.18	10.75±0.23^a^	9.28±0.17	8.69±0.17	9.24±0.23
GSH (µmol/ml)	159.31±5.88	148.74±8.02	149.59±6.04	149.04±5.76	117.31±3.78^a^	142.82±9.99	143.88±5.39	143.46±7.47
CAT (k/g Hb)	30.61±1.85	27.40±1.69	34.97±1.76	26.84±2.00	19.98±0.80^a^	27.19±2.27	26.79±0.96	29.08±2.20
GPx (U/g Hb)	113.25±1.29	114.73±2.62	114.61±6.15	122.01±7.62	80.38±3.68^a^	112.71±11.67	107.40±3.95	112.34±4.69
SOD (U/g Hb)	63.01±0.48	63.03±0.51	62.43±0.61	62.89±0.61	58.10±0.77^a^	60.77±0.99	62.44±0.63	62.91±0.54
TAS (U/mL)	4.65±0.16	4.65±0.35	4.78±0.38	4.67±0.24	4.20±0.25^a^	4.55±0.25	4.32±0.09	4.60±0.13
TOS (nmol/mL)	2.27±0.16	2.31±0.07	2.28±0.18	2.34±0.20	2.36±0.22	2.30±0.19	2.29±0.32	2.30±0.37

**Table 3:** MDA, GSH, CAT, GPx, SOD, TAS, and TOS levels in the liver tissues of rats exposed to the control group, O3 group, TQ group, TQ+O3 group, FA group, FA+O3 group, FA+TQ group, and FA+TQ+O3 group. ^a^Significantly different compared to the control group. MDA: malondialdehyde; GSH: glutathione reductase; CAT: catalase; GPx: glutathione peroxidase; SOD: superoxide dismutase; TAS: total antioxidant level; TOS: total oxidant level; TQ: thymoquinone; O_3_: ozone; FA: formaldehyde.

	Control	O_3_	TQ	TQ+O_3_	FA	FA+O_3_	FA+TQ	FA+TQ+O_3_
MDA (nmol/ml)	0.65±0.03	0.54±0.06	0.62±0.05	0.58±0.03	0.96±0.06^a^	0.77±0.05	0.78±0.05	0.71±0.0
GSH (µmol/ml)	14.67±0.49	17.54±0.18	16.64±0.30	17.13±0.21	14.93±0.35^a^	16.64±0.32	17.17±0.25	16.73±0.23
CAT (k/g Hb)	34.22±2.51	33.65±2.87	40.05±1.94	37.22±1.72	25.57±1.76^a^	35.07±2.22	34.02±2.47	35.07±2.22
GPx (U/g Hb)	18.45±0.57	16.49±0.57	18.18±0.53	16.97±0.55	14.30±0.58^a^	18.39±1.02	17.23±0.47	17.58±0.41
SOD (U/g Hb)	12.99±0.05	12.80±0.10	13.25±0.19	13.43±0.27	11.91±0.14^a^	12.93±0.10	13.00±0.18	12.92±0.12
TAS (U/mL)	8.04±0.42	8.16±0.84	8.19±0.33	8.25±8.40	7.63±0.37	7.83±0.41	7.93±0.34	8.09±0.32
TOS (nmol/mL)	2.76±0.19	2.74±0.38	2.75±0.48	2.75±0.45	2.92±0.66^a^	2.81±0.46	2.82±0.32	2.85±0.12

Compared to the control group, there was no significant difference in MDA, GSH, CAT, GPx, SOD, TAS, and TOS levels in serum and liver tissue in the O_3_ group, TQ group, and TQ+O_3_ group (Tables 2, 3).

Compared with the control group, serum MDA levels were significantly increased (p=0.001), and GSH, CAT, GPx, SOD, and TAS levels were significantly decreased (GSH, CAT: p<0.05; GPx, SOD: p=0.001; TAS: p=0.03) in the FA group. There was no significant difference in serum TOS levels compared to the control group. Compared with the control group in the liver tissue, MDA levels were significantly increased (p=0.001), and GSH, CAT, GPx, and SOD were significantly decreased (GSH, CAT: p<0.05; GPx, SOD: p=0.001) in the FA group. However, there was no significant difference in TAS and TOS levels (Tables 2, 3).

In the FA+TQ group, serum MDA levels were significantly decreased, GSH, CAT, GPx, and SOD levels were significantly increased, TAS levels were not significantly increased, and TOS levels were not significantly decreased compared to the FA group. Furthermore, the serum TAS level was significantly decreased in the FA+TQ group compared to the control group; however, there was no difference in other parameters. In addition, there was no significant difference in MDA, GSH, CAT, GPx, SOD, TAS, and TOS levels in liver tissue between the FA+TQ group and the control group (Tables 2, 3).

Comparing the FA+O_3_ group with the control group, serum SOD level was significantly decreased, but there was no difference in other parameters. MDA levels were significantly decreased, while GSH, CAT, GPx, SOD, and TAS levels were significantly increased in this group compared to the FA group. TOS levels were not significantly decreased in this group. No significant difference was found in MDA, GSH, CAT, GPx, SOD, TAS, and TOS levels in liver tissue between the FA+O3 group and the control group (Tables 2, 3).

MDA levels were significantly decreased in the FA+O_3_+TQ group compared to the FA group, while GSH, CAT, GPx, SOD, and TAS levels were significantly increased. However, there was an insignificant decrease in TOS levels. Furthermore, no significant difference was found in serum MDA, GSH, CAT, GPx, SOD, TAS, and TOS levels in the FA+O_3_+TQ group compared to the control group. It was also determined that there was no significant difference between the FA+TQ+O_3_ group and the control group in MDA, GSH, CAT, GPx, SOD, TAS, and TOS levels in liver tissue (Tables 2, 3).

Immunohistochemical findings

CD68 Immunoreactivity in Liver Tissue

Immunohistochemical staining for CD68 immunoreactivity under a light microscope revealed that CD68 immunoreactivity was only observed in Kupffer cells (black arrow) in liver tissue. Compared to the control group, CD68 immunoreactivity was similar in the TQ (p=0.097) and TQ+O_3_ (p=0.318) groups, while it was statistically significantly increased in the O_3_ group (p<0.001). In addition, CD68 immunoreactivity was significantly decreased in the FA group (p=0.004) compared to the control group.

**Figure 1:**
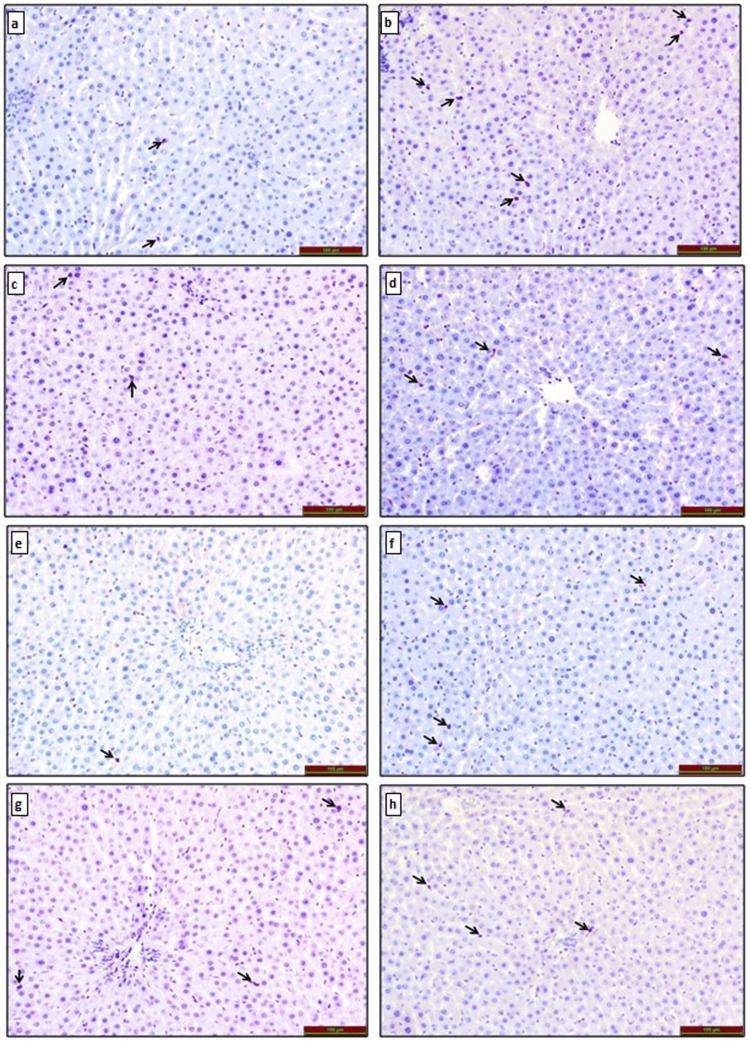
CD68 immunoreactivity in liver tissues of rats exposed to (a) control group, (b) O3 group, (c) TQ group, (d) TQ+O3 group, (e) FA group, (f) FA+O3 group, (g) FA+TQ group, and (h) FA+TQ+O3 group. CD68 immunoreactivity is indicated by the black arrow in the liver. TQ: thymoquinone; O_3_: ozone; FA: formaldehyde.

Compared to the FA group, CD68 immunoreactivity was significantly increased in the FA+O_3_ (p<0.001), FA+TQ (p=0.004), and FA+O_3_+TQ (p=0.002) groups. Among the treatment groups, a statistically significant decrease in CD68 immunoreactivity was observed in the FA+TQ (p<0.001) and FA+O_3_+TQ (p<0.001) groups compared to the FA+O_3_ group (Figure 1).

TUNEL Staining in Liver Tissue

TUNEL staining was used to identify apoptotic cells, which were examined under a light microscope. Compared to the control group, TUNEL positivity was similar in the O_3_ (p=0.209), TQ (p=0.805), and TQ+O_3_ (p=0.620) groups, while TUNEL positivity was statistically significantly increased in the FA group (p<0.001).

**Figure 2:**
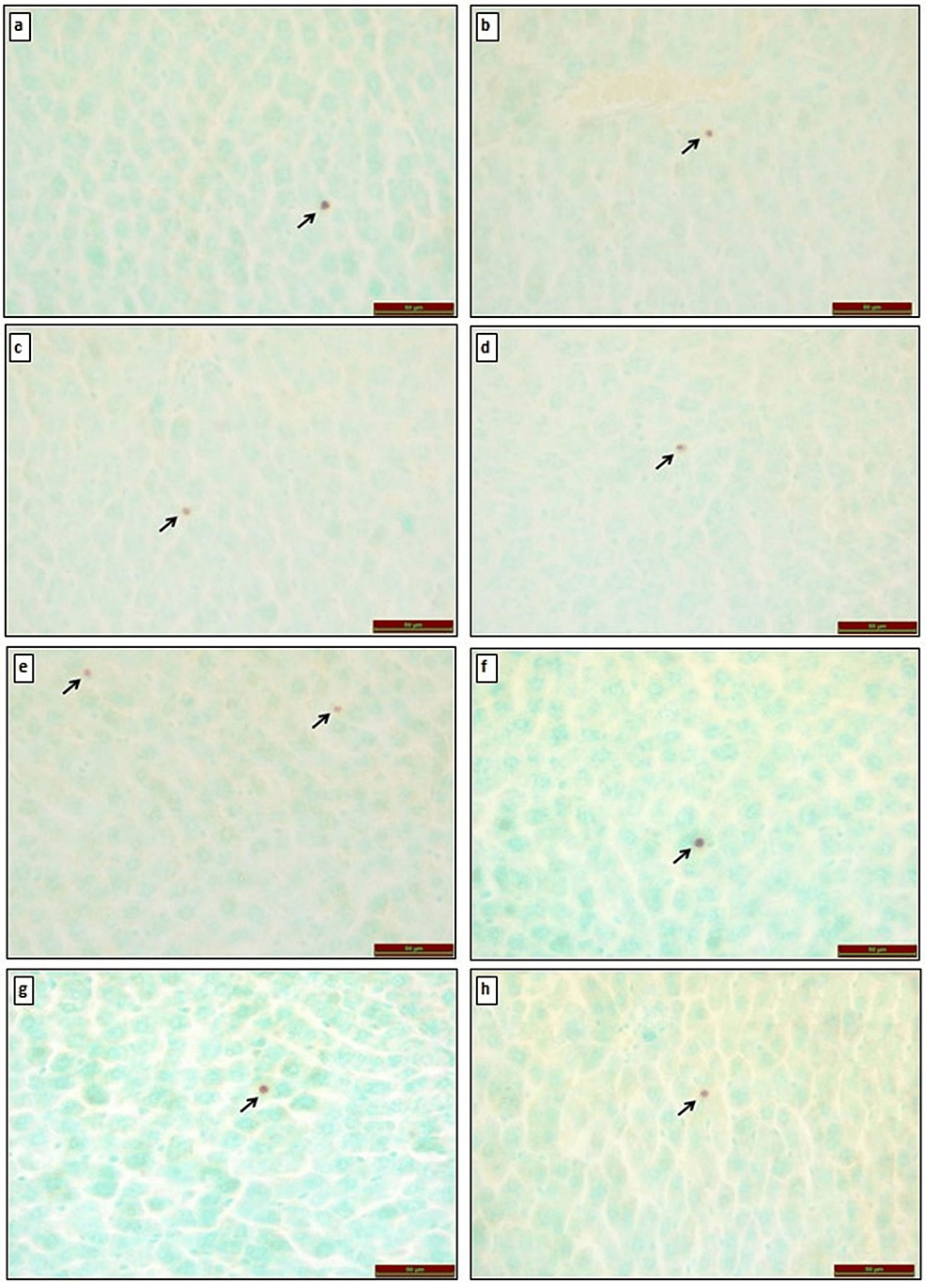
TUNEL alterations in liver tissues of rats exposed to (a) control group, (b) O3 group, (c) TQ group, (d) TQ+O3 group, (e) FA group, (f) FA+O3 group, (g) FA+TQ group, and (h) FA+TQ+O3 group. Apoptotic cells in the liver are indicated by black arrows. TUNEL: terminal deoxynucleotidyl transferase dUTP nick end labeling; TQ: thymoquinone; O_3_: ozone; FA: formaldehyde.

In comparison with the FA group, TUNEL positivity was significantly decreased in the FA+O_3_ (p<0.001), FA+TQ (p<0.001), and FA+O_3_+TQ (p<0.001) groups (Figure 2).

## Discussion

When TQ was used alone in the treatment of toxic exposure to FA, there was a significant decrease in serum MDA levels, a significant increase in GPx, GSH, CAT, and SOD levels, a non-significant increase in TAS levels, and a non-significant decrease in TOS levels. When ozone therapy was used alone, MDA levels decreased significantly, GSH, CAT, GPx, SOD, and TAS levels increased significantly, and TOS levels decreased insignificantly. When ozone therapy was used in combination with TQ, there was a significant decrease in MDA levels and a significant increase in GSH, CAT, GPx, SOD, and TAS levels. However, an insignificant decrease was observed in TOS levels.

Enzymatic and non-enzymatic defenses in the body attempt to block oxidant attacks. The core of all enzymatic defenses is a transition metal that can take on different values when transferring electrons during the detoxification process. Cells synthesize glutathione, the primary thiol redox buffer, from L-glutamate, L-cysteine, and glycine in the cytosol. Glutathione reductase catalyzes the reduction of glutathione disulfide to the sulfhydryl form of glutathione (GSH), a molecule critical in resistance to oxidative stress and maintaining the cell's reducing environment [17]. Endogenous and exogenous free radicals cause oxidative stress. The condition occurs when the oxidant and antioxidant balance in the body is disrupted in favor of oxidants. The total value of oxidative stress is expressed as TOS. Excessive formation of reactive oxygen radicals or inadequacy of the antioxidant buffer system leads to increased TOS values. Reactive oxygen radicals have been reported to damage the lipid and protein structures and the cell's DNA. TAS refers to the total antioxidant protection in the organism against attack by free radicals. Measurement of TAS level provides more significant information than measurement of antioxidants individually [18].

In animal studies, it has been reported that FA inhalation increases free radicals, disrupts the oxidant-antioxidant balance, and increases oxidation capacity through changes in the activities of antioxidant enzymes such as SOD, GSH, GPx, MDA, and CAT [19,20]. In this study, TOS elevated with exposure to FA. We hypothesize that this increase is caused by free radicals, as previously reported in other studies [21,22]. TQ and ozone therapy appear to have a restorative effect on the oxidant/antioxidant balance disrupted by exposure to FA. Although studies supporting this are available in the literature [23-26], no studies have investigated using two antioxidants together.

No data on the relationship between FA exposure, TQ, ozone therapy, and OSI were found in the literature. In addition, several studies have shown that OSI is associated with total antioxidant and oxidant capacity [27].

To show the effect of FA on cell death and apoptosis, TUNEL staining was performed. Apoptosis increased with exposure to FA and decreased with TQ and ozone therapy. Separate studies have reported that TQ and ozone therapy suppress apoptosis [24,28-30]. No studies in the literature have examined the combined effects of two antioxidants against exposure to FA.

## Conclusions

FA exposure damages liver tissues and increases AST and ALT in the blood. Exposure to FA decreases GSH, CAT, GPx, SOD, and TAS levels and increases TOS levels and TUNEL reactivity in both serum and tissue. TQ and ozone therapy are antioxidants that may be beneficial against the harmful effects of FA exposure. Nowadays, ozone therapy is an alternative treatment method. In our study, it was determined that ozone therapy was supportive and did not interact negatively with TQ, another antioxidant. As a result of more comprehensive studies conducted in the future, TQ and ozone therapy may be among the protective agents against the harmful effects of FA exposure.
